# A plasmonic colorimetric strategy for visual miRNA detection based on hybridization chain reaction

**DOI:** 10.1038/srep32219

**Published:** 2016-08-18

**Authors:** Jie Miao, Jingsheng Wang, Jinyang Guo, Huiguang Gao, Kun Han, Chengmin Jiang, Peng Miao

**Affiliations:** 1 Department of Clinical Laboratory, the 404th Hospital of PLA, Weihai 264200, P. R. China; 2 CAS Key Lab of Bio-Medical Diagnostics, Suzhou Institute of Biomedical Engineering and Technology, Chinese Academy of Sciences, Suzhou 215163, P. R. China; 3 Department of Chemistry, Rice University, Houston, Texas 77005, United States

## Abstract

In this work, a novel colorimetric strategy for miRNA analysis is proposed based on hybridization chain reaction (HCR)-mediated localized surface plasmon resonance (LSPR) variation of silver nanoparticles (AgNPs). miRNA in the sample to be tested is able to release HCR initiator from a solid interface to AgNPs colloid system by toehold exchange-mediated strand displacement, which then triggers the consumption of fuel strands with single-stranded tails for HCR. The final produced long nicked double-stranded DNA loses the ability to protect AgNPs from salt-induced aggregation. The stability variation of the colloid system can then be monitored by recording corresponding UV-vis spectrum and initial miRNA level is thus determined. This sensing system involves only four DNA strands which is quite simple. The practical utility is confirmed to be excellent by employing different biological samples.

miRNAs are a group of single-stranded, endogenous, small noncoding RNA molecules (approximately 18–25 nucleotides), which play significant regulatory roles in numerous biological processes including cellular proliferation, differentiation and apoptosis^1^,^2^,^3^. Aberrant expressions of certain miRNAs have been found to be indicative in a variety of human diseases^4^. Recently, certain miRNAs have been employed as biomarkers for early clinical diagnosis of some cardiovascular diseases, infectious diseases and cancers^5^,^6^,^7^,^8^. For instance, miR-29a-3p is found to be down-regulated in throat swabs of the subjects infected with influenza A virus H1N1^9^; let-7a levels in severe asthma patients can be used to distinguish distinct phenotypes of the disease^10^; mir-133b, mir-133a-2, and mir-1-2 levels are found to be significantly correlated with tumor pathologic and stage of gastric cancer^11^. So far, great efforts have been made for the development of sensitive and selective analytical methods. However, some obstacles exist and traditional techniques are limited for certain applications^12^,^13^. For example, qRT-PCR requires complicated primer design and housekeeping gene for normalization^14^; microarray is able to realize fluorescent imaging of miRNAs with multiple channels but only semi-quantitative information can be obtained^15^; electrochemical techniques always need time-consuming modification of electrode interface^16^; surface enhanced Raman spectroscopy (SERS) is rarely used in application for non-SERS active molecules^17^. Hence, it is important to establish advanced methods for miRNA quantification with simple design and high sensitivity.

The field of plasmonic colorimetric sensing based on noble metal nanoparticles has been well established with high sensitivities, and convenient operations without complicated labels^18^,^19^,^20^,^21^,^22^. In recent years, different silver nanoparticles (AgNPs) synthesis and modification methods have been developed for the detection of a variety of molecules based on the localized surface plasmon resonance (LSPR) effect^23^,^24^,^25^. These studies exhibit certain advantages due to fine properties of AgNPs like easy of synthesis, high extinction coefficients and stability^26^,^27^.

Herein, we propose a novel hybridization chain reaction (HCR)-based colorimetric biosensor to conveniently detect miR-29a-3p for the application of clinical diagnosis. HCR is an enzyme-free and target-induced fuel strands assembly process^28^. The final product is long nicked double-stranded DNA. The reaction has been widely applied for DNA detection with PCR-like sensitivity^29^,^30^,^31^. In this work, we have realized the release of HCR initiator from a solid interface by target miRNA, which triggers HCR in AgNPs colloid system in the presence of two DNA probes. Since single-stranded DNA interacts with AgNPs through the exposed nitrogen-containing bases, nanoparticles aggregation induced by salt can be effectively prevented. Therefore, single-stranded DNA can act as the stabilizer of the colloid system. Nevertheless, double-stranded DNA exposes no nitrogen-containing bases and cannot interact with AgNPs. After HCR initiated by target miRNA, the transformation of fuel strands with single-stranded DNA tails to double-stranded DNA changes the stabilization ability of DNA molecules in AgNPs colloid system, which can be reflected by UV-vis spectrum. This simply designed method for the detection of miRNA achieves the limit of detection as low as 1 pM, which is lower than or at least comparable to other enzyme-mediated colorimetric assays.

## Results and Discussion

**Figure 1:**
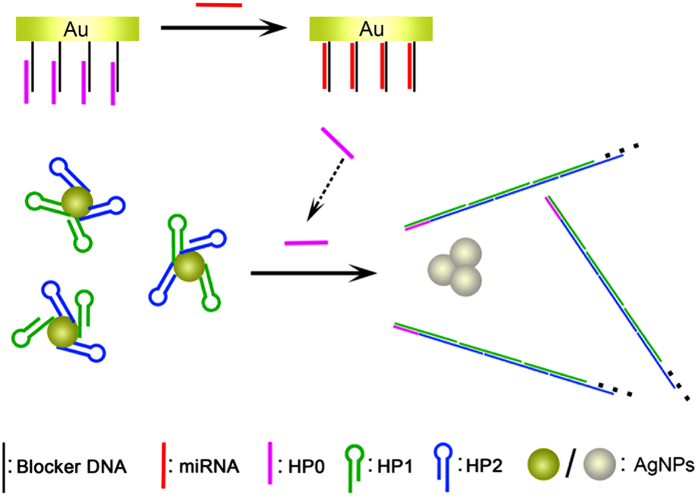
Scheme of the colorimetric strategy for miRNA assay.

The detailed strategy for colorimetric assay of miRNA is illustrated in Fig. 1. Briefly, Blocker DNA is immobilized on a gold solid interface via thiol-gold interaction^32^. By partial hybridization with another DNA probe named HP0, double-stranded DNA is formed. The gold solid interface is then immersed in the mixture of AgNPs and two DNA probes named as HP1 and HP2, which are not only two fuels strands for hybridization chain reaction^33^, but also act as stabilizers of AgNPs colloid system. The presence of HP1 and HP2 makes AgNPs much more stable. The nanoparticles disperse well in 0.1 M MgCl_2_ and the UV–vis spectrum peak barely changes. Since a toehold exists at the duplex on the gold solid interface, the introduced target miRNA could initiate strand displacement reaction, releasing HP0 back to the solution, which further triggers the hybridization chain reaction, generating long double-stranded DNA product. Thereby, due to the lack of single-stranded tails of HP1 and HP2, the colloid system of AgNPs is less stable against the treatment of salt. By analyzing UV-vis spectrum of the system, the phenomenon of miRNA-induced aggregation of AgNPs could be quantified.

**Figure 2:**
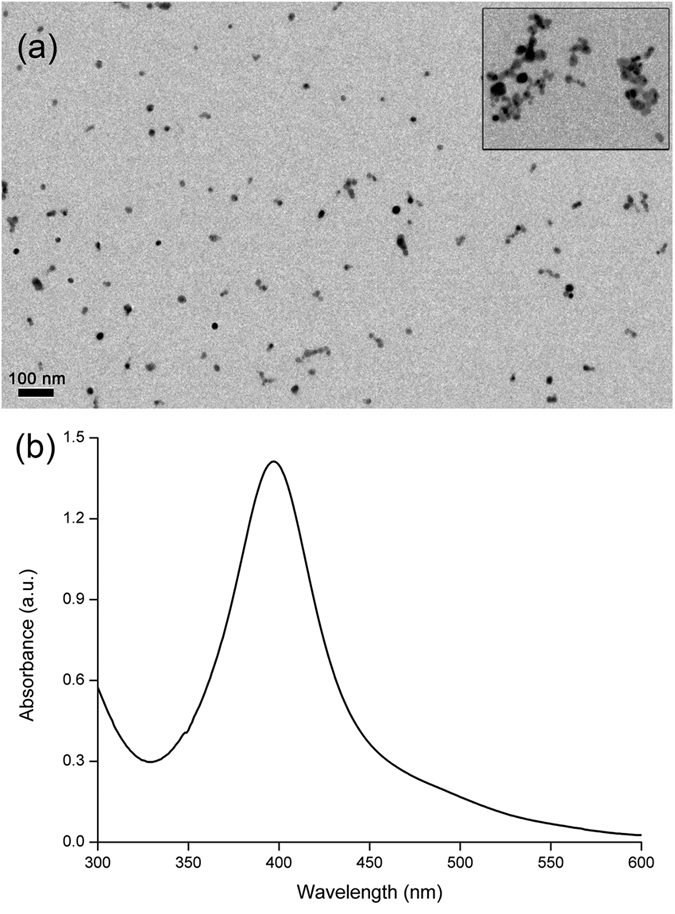
(**a**) TEM image of AgNPs. Inset is the image after miRNA induced HP0 release and subsequent HCR. (**b**) UV–vis spectrum of bare AgNPs.

The prepared AgNPs suspension is transparent and yellow colored. From the TEM image in Fig. 2a, it is observed that AgNPs disperse well in water and the diameter is about 5 nm. Moreover, a well characteristic absorbance peak at the wavelength around 400 nm can be confirmed in the UV–vis spectrum of AgNPs (Fig. 2b). After miRNA-induced HP0 release and the following HCR, the aggregation of AgNPs can be observed, which confirms the effectiveness of the proposed strategy (inset in Fig. 2a).

**Figure 3:**
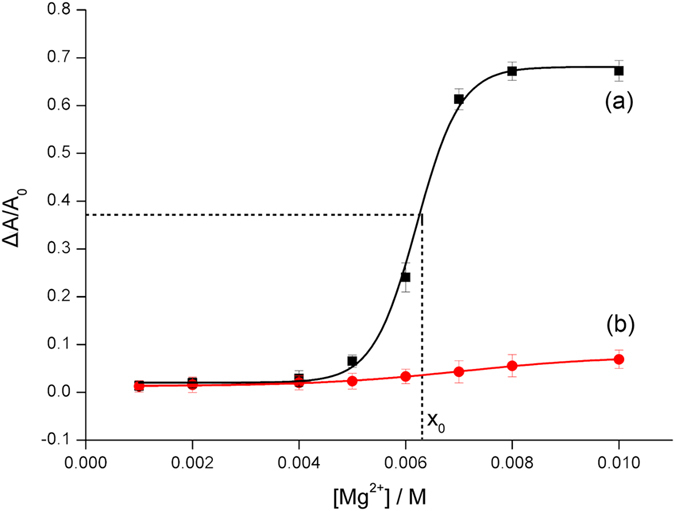
ΔA/A_0_ of (**a**) bare AgNPs, (**b**) HP1 and HP2 protected AgNPs versus the concentration of Mg^2+^. The slim dash lines represent the position of the critical point for salt-tolerance of bare AgNPs. Error bars represent standard deviations of three measurements.

To achieve the optimized aggregation condition, we have performed preliminary experiments. Divalent cations are much more effective to neutralize negative charges on the surface of AgNPs than monovalent cations like Na^+^. A series of Mg^2+^ concentrations have been used to check the salt-tolerance of AgNPs. More Mg^2+^ may lead to the larger decrease of the absorbance peak. The ratio of net decreased peak (ΔA/A_0_) is used to represent the distribution state of AgNPs. Figure 3a shows the relationship between ΔA/A_0_ of bare AgNPs and the concentration of Mg^2+^. The fitting curve is a Boltzmann sigmoid and the equation is as follows:


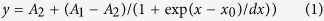


in which *y* is ΔA/A_0_, *x* is the concentration of Mg^2+^, *A*_*1*_ = 0.0204, *A*_*2*_ = 0.6810, *x*_*0*_ = 0.0062, *dx* = 0.0004, *n* = 3, *R*^*2*^ = 0.9977. The slope of this fitting curve reaches a maximum value at the point of 0.0062, indicating that AgNPs is most sensitive to the changes of salt when the concentration of Mg^2+^ is 6.2 mM, which is then used for the following colorimetric investigation. However, in the case of AgNPs protected by HP1 and HP2, ΔA/A_0_ barely increases after the treatment of Mg^2+^, which demonstrates the high stability of the AgNPs stabilized by DNA probes with single-stranded DNA tails (Fig. 3b).

**Figure 4:**
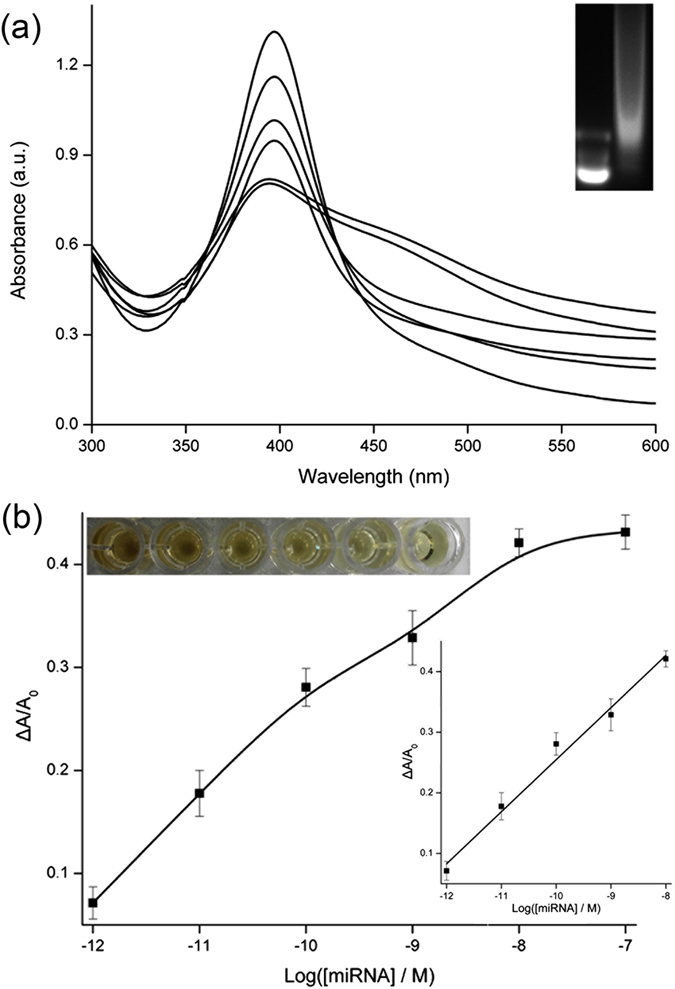
(**a**) UV–vis spectra of mixtures of AgNPs, Mg^2+^, HP1, HP2, and HP0 released by miRNA with different concentrations: 1 pM, 10 pM, 100 pM, 1 nM, 10 nM, 100 nM (from top to bottom). Inset is the agarose gel electrophoresis analysis of mixture of HP1 and HP2 (left) and mixture of HP0, HP1 and HP2 (right). (**b**) Calibration curve of ΔA/A_0_ versus logarithmic miRNA concentration. Inset are the corresponding solution colors and linear curve.

**Figure 5:**
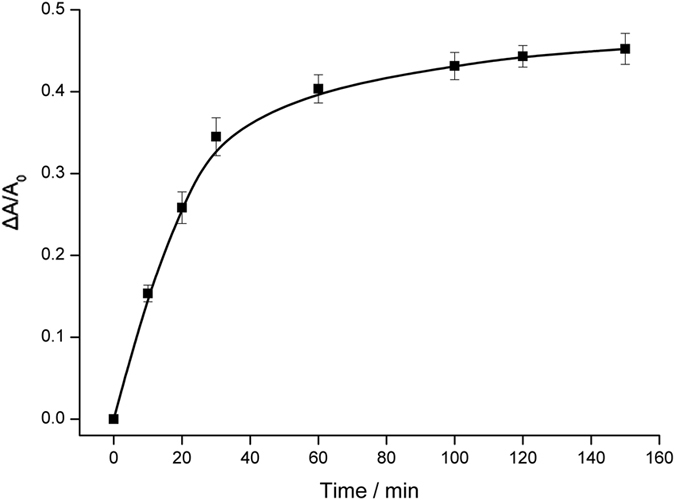
Relationship between ΔA/A_0_ and duration of hybridization chain reaction.

UV-vis spectra are recorded for the experiments using miRNA of different concentrations to trigger strand displacement reaction and further HCR. HCR could be directly confirmed by a gel electrophoresis experiment. The bands representing much larger molecular weight are observed after the reaction, indicating the formation of assembled long double-stranded DNA (inset in Fig. 4a). Furthermore, the presence of miRNA leads to the decrease of absorbance peak, which is shown in Fig. 4a. More miRNA releases more HP0, and leads to the consumption of more stabilizers. Finally, the absorbance peak decreases accordingly. Optimized reaction time of hybridization chain reaction is identified to be 100 min by comparing the recorded absorbance peaks (Fig. 5). Figure 4b depicts the calibration curve reflecting the relationship between ΔA/A_0_ and miRNA concentration. Corresponding AgNPs colors are also shown. From the Figure, a linear dependency is obtained with a fitting equation of


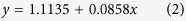


in which *y* is ΔA/A_0_, *x* is the concentration of target miRNA, *n* = 3, *R*^*2*^ = 0.9869. The limit of detection is calculated to be 1 pM (S/N = 3), which is sufficient for the application of clinical diagnosis. For example, influenza A virus H1N1 diagnostics can be performed by the detection of the biomarker of miR-29a-3p using the proposed colorimetric strategy.

A representative member of miR-29 family, miR-29c, is chosen to be detected by the proposed method, which contains only single base mismatch compared with miR-29a. The resulted ΔA/A_0_ is negligible, which confirms the high specificity of this method for the detection or target miRNA.

In addition, we have carried out experiments involving real samples to check the practical utility of this colorimetric biosensor. Throat swabs from healthy and infected individuals are collected and treated before the colorimetric assay. Moreover, a commercial qRT-PCR Kit is employed to obtain the control values. The results listed in Table 1 reveal that the detected values of the two methods are consistent with each other. The proposed colorimetric assay could distinguish infected individuals by accurately determining the level of the biomarker. Sampling and colorimetric assay operations are both quite simple, which could promise great practical utility.

In summary, we have herein developed a novel colorimetric strategy for visual miRNA detection based on hybridization chain reaction. Target miRNA could release HP0 to the solution to trigger HCR, which hinders the protection of AgNPs by single-stranded DNA tails of HP1 and HP2 from divalent cation-induced aggregation. The information of UV–vis spectra can then be used to reveal the initial miRNA level. This non-enzymatic method involves only four DNA strands, which is quite simple. Moreover, it also has high sensitivity towards the clinical diagnosis applications.

## Methods

### Materials and Chemicals

The methods were carried out in accordance with institutional and national guidelines and with the approval of the Medical Ethics Committee of The First Affiliated Hospital of Soochow University (no.2012076). Informed consent was obtained from all subjects. The study was also conducted in accordance with the Declaration of Helsinki. Silver nitrate (AgNO_3_), sodium borohydride (NaBH_4_), tris(2-carboxyethyl) phosphine hydrochloride (TCEP), trisodium citrate, diethypyrocarbonate (DEPC), mercaptohexanol (MCH), ethylenediaminetetraacetic acid (EDTA) were purchased from Sigma (USA). All other reagents were of analytical grade. Human samples were from local hospital. Water used was previously purified by a Millipore system to 18 MΩ cm resistivity and treated with DEPC. All DNAs and miRNA were obtained from Takara Biotechnology Co., Ltd. (Dalian, China). The sequence were listed in Table 2. TEM images were taken by using a FEI Tecnai G20 transmission electron microscopy (FEI, USA). UV-vis spectra were measured by an Agilent Cary 300 Scan UV-vis absorption spectrophotometer (Agilent Technologies, USA).

### Preparation of AgNPs

Bare AgNPs were synthesized according to a previously reported protocol^34^. Briefly, AgNO_3_ solution and trisodium citrate solution were prepared and mixed. The concentrations of AgNO_3_ and trisodium citrate were both 0.25 mM. NaBH_4_ solution was then prepared with the concentration of 10 mM. 3 mL of NaBH_4_ solution was added to 100 mL of the mixture of AgNO_3_ and trisodium citrate. Then, the reaction solution was stirred vigorously for 0.5 h. Afterwards, the solution was kept in dark and left to sit for 24 h. The formed AgNPs were later purified by three cycles of centrifugation at 12,000 g for 0.5 h.

### Optimization of Mg^2+^ Concentration

Bare AgNPs could keep stable due to presence of negatively charged citrate. However, with the disturbance of salt in the colloid system, aggregation occurred and the color of the solution changed correspondingly. Mg^2+^ with a series of concentrations were prepared to induce the aggregation of AgNPs. Absorbance peaks were recorded and ratios of net decreased peak values (ΔA/A_0_) were calculated. After analysing the data, the critical value of Mg^2+^ to induce aggregation was found to represent the Mg^2+^-tolerance of AgNPs^35^.

### Gel electrophoresis Experiments

Different DNA samples were monitored for comparison by 4% agarose gel electrophoresis for 30 min (100 V). The photograph of gel stained with ethidium bromide was taken under UV light by a Gel DocTM XR+ System (Bio-Rad, USA).

### UV-vis Spectroscopy Analysis of miRNA

Plasmonic colorimetric detection of miRNA was carried out as follows. 2 μM HP1 and HP2 were added to AgNPs solution to protect the colloid system. Standard miRNA solutions with the concentration from 1 pM to 100 nM was prepared to treat Blocker DNA/HP0 duplex modified gold solid interface for 1 h. The released HP0 in the solution was then transferred to HP1 and HP2 protected AgNPs. Since HP0 could initiate HCR and make colloid system less stable, after the addition of Mg^2+^ with the critical value, AgNPs aggregated, which was recorded by UV-vis absorption spectrophotometer.

### Determination of miRNA in Real Samples

To verify the practical utility of this method in human samples, throat swabs from healthy and infected (H1N1 influenza A virus) individuals were collected and diluted by sterile saline for miRNA analysis. Total RNA were extracted by a RNA extraction kit from Qiagen according to manufacture’s instructions. The levels of miR-29a-3p were then calculated referring to the linear curve of the proposed colorimetric assay. In addition, the results obtained from commercial Quant One Step qRT-PCR Kit were used as the controls.

## Additional Information

**How to cite this article**: Miao, J. *et al.* A plasmonic colorimetric strategy for visual miRNA detection based on hybridization chain reaction. *Sci. Rep.* **6**, 32219; doi: 10.1038/srep32219 (2016).

## Figures and Tables

**Table 1:** Validations of miR-29a-3p levels in samples from healthy and infected individuals by the proposed colorimetric method and qRT-PCR.

Sample	Colorimetry (pM)	qRT-PCR (pM)
Healthy individual	83	86
	75	72
Infected individual	17	19
	14	18

**Table 2:** DNA and miRNA sequences.

Name	Sequence (from 5′ to 3′)
miR-29a-3p	UAGCACCAUCUGAAAUCGGUUA
miR-29c-3p	UAGCACCAU**U**UGAAAUCGGUUA
Blocker DNA	CCGTAACCGATTTCAGATGGTGCTATTTT-SH
HP0	CGGTTACGGTTACGGTTA
HP1	CGGTTACGGTTACTAGAGTAACCGTAACCGTAACCG
HP2	CTCTAGTAACCGTAACCGCGGTTACGGTTACGGTTA
